# Longitudinal monitoring by next‐generation sequencing of plasma cell‐free DNA in ALK rearranged NSCLC patients treated with ALK tyrosine kinase inhibitors

**DOI:** 10.1002/cam4.4663

**Published:** 2022-04-19

**Authors:** Minsuk Kwon, Bo mi Ku, Steve Olsen, Sehhoon Park, Martina Lefterova, Justin Odegaard, Hyun‐Ae Jung, Jong‐Mu Sun, Se‐Hoon Lee, Jin Seok Ahn, Keunchil Park, Myung‐Ju Ahn

**Affiliations:** ^1^ Division of Hematology‐Oncology, Department of Medicine, Samsung Medical Center Sungkyunkwan University School of Medicine Seoul Republic of Korea; ^2^ Department of Hematology‐Oncology Ajou University School of Medicine Suwon South Korea; ^3^ Department of Medical and Clinical Affairs Guardant Health AMEA Singapore Singapore

**Keywords:** anaplastic lymphoma kinase‐rearranged (*ALK+*) NSCLC, cell‐free DNA, liquid biopsy, next‐generation sequencing

## Abstract

**Background:**

Patients with *ALK‐*rearranged non‐small cell lung cancer (*ALK+* NSCLC) inevitably acquire resistance to ALK inhibitors. Longitudinal monitoring of cell‐free plasma DNA (cfDNA) next‐generation sequencing (NGS) could predict the response and resistance to tyrosine kinase inhibitor (TKI) therapy in *ALK+* NSCLC.

**Methods:**

Patients with *ALK+* NSCLC determined by standard tissue testing and planned to undergo TKI therapy were prospectively recruited. Plasma was collected at pretreatment, 2 months‐post therapy, and at progression for cfDNA‐NGS analysis, Guardant 360.

**Results:**

Among 92 patients enrolled, circulating tumor DNA (ctDNA) was detected in 69 baseline samples (75%): 43 *ALK* fusions (62.3%) and two *ALK* mutations without fusion (2.8%). Two patients showed *ALK*‐resistance mutations after ceritinib; G1202R, and co‐occurring G1202R and T1151R. Eight patients developed *ALK* resistance mutations after crizotinib therapy; L1196M (*n* = 5), G1269A (*n* = 1), G1202R (*n* = 1), and co‐occurring F1174L, G1202R, and G1269A (*n* = 1). Absence of ctDNA at baseline was significantly associated with longer progression‐free survival (PFS; median 36.1 vs. 11.4 months, *p* = 0.0049) and overall survival (OS; not reached vs. 29.3 months, *p* = 0.0200). ctDNA clearance at 2 months (*n* = 29) was associated with significantly longer PFS (25.4 vs. 11.6 months, *p* = 0.0012) and OS (not reached vs. 26.1 months, *p* = 0.0307) than those without clearance (*n* = 22). Patients with co‐occurring *TP53* alterations and *ALK* fusions at baseline (*n* = 16) showed significantly shorter PFS (7.28 vs. 13.0 months, *p* = 0.0307) than those without *TP53* alterations (*n* = 25).

**Conclusions:**

cfDNA‐NGS facilitates detection of *ALK* fusions and resistance mutations, assessment of prognosis, and monitoring dynamic changes of genomic alterations in *ALK*+ NSCLC treated with ALK‐TKI.

## Graphical abstract

To investigate whether longitudinal monitoring of cfDNA‐NGS could predict the response and resistance of TKI therapy in ALK+ NSCLC, we recruited prospectively 92 patients with ALK+ advanced NSCLC determined by standard tissue testing and planned for TKI therapy. NGS of cfDNA is useful not only for the detection of ALK fusions and resistance mutations, but also for assessing prognosis and monitoring the dynamic changes of genomic alterations in ALK+ NSCLC treated with ALK‐TKI.

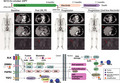

## 1 INTRODUCTION

Rearrangement of anaplastic lymphoma kinase gene (ALK) is oncogenic driver which accounts for 3%–5% of patients with non‐small cell lung cancer (NSCLC).^1^, ^2^ During the last decade, significant efforts from academia and the pharmaceutical industry have led to the development of numerous ALK tyrosine kinase inhibitors (TKI). Since the first‐generation ALK inhibitor crizotinib^3^ was the standard therapy for newly diagnosed *ALK* positive NSCLC, more potent CNS penetrating second generation ALK inhibitors such as ceritinib,^4^ alectinib,^5^ brigatinib,^6^ and lorlatinib^7^ have become the standard treatment as first‐line therapy.

Remarkable therapeutic efficacies were observed with these ALK TKIs, however, essentially many patients inevitably develop acquired resistance within 1 or 2 years. The resistance mechanisms of ALK TKI are categorized into “on‐target” mechanisms such as *ALK* secondary resistance mutation or amplification, where oncogenic dependencies on ALK kinase persist, and “off‐target” mechanisms such as activation of bypass pathway or lineage changes, where the cancer cells can survive and proliferate regardless of ALK activity.^8^ Of note, the incidence and spectrum of secondary mutations in the ALK tyrosine kinase domain is variable among ALK TKIs. L1196M or G1269R is found in 4 ~ 7% of patients resistant to crizotinib, whereas G1202R mutation is observed in 20%–40% of patients resistant to ceritinib, alectinib, or brigatinib.^9^ Furthermore, several ALK secondary mutations represent differential sensitivity to the type of ALK TKIs.^9^ Therefore, the elucidation of resistance mechanisms is critical to decide subsequent treatment strategies in *ALK* positive NSCLC who experience progression to ALK TKIs.

Comprehensive next‐generation sequencing (NGS) tests covering all relevant genomic biomarkers by repeat tissue biopsy may be preferred to determine the resistance mechanism. However, tissue NGS tests have challenges regarding tissue availability, quality, and long turn‐around time. Considering intra‐tumoral or inter‐tumoral heterogeneity, small tissue biopsy may not represent the overall genomic status of tumor. To overcome these limitations, NGS of cell‐free plasma DNA (cfDNA‐NGS) has gained substantial attention as an alternative strategy.^10^, ^11^, ^12^ Further, liquid biopsy can make it possible to perform longitudinal monitoring during ALK TKI treatment as noninvasive approach.^13^

Technological advances in cfDNA‐NGS has broadened the clinical use in various solid tumors.^14^, ^15^, ^16^ Prior work reported that cfDNA‐NGS test was applicable and useful in *ALK* positive NSCLC patients,^17^, ^18^, ^19^, ^20^, ^21^, ^22^ but the small number of patients studied still remain a limitation to determine the predictive role of cfDNA‐NGS in the *ALK* positive NSCLC patients who were being treated with TKI.

This study was initiated to evaluate the role of cfDNA‐NGS not only for the detection of *ALK* fusions and resistance mutations, but also for assessing prognosis and monitoring the dynamic changes of genomic alterations in *ALK* positive NSCLC patients treated with ALK TKIs in prospective manner.

## 2 PATIENTS AND METHODS

### 2.1 Study population

Patients diagnosed with *ALK* rearranged advanced or recurrent NSCLC who were treated with ALK TKIs were enrolled. Patients previously treated with cytotoxic chemotherapy or ALK TKI therapy were not excluded from the study. The choice of ALK TKI was made by the physician's discretion. The *ALK* positive NSCLC was diagnosed by ALK immunohistochemical stain (IHC) using the Ventana anti‐ALK (D5F3), FISH, or NGS. Tumor responses were evaluated every two cycles (8 weeks) according to Response Evaluation Criteria in Solid Tumors (RECIST version 1.1). This study protocol was reviewed and approved by the institutional review boards of Samsung Medical Center (SMC 2013_09‐075, Seoul, Korea). Written informed consent was obtained from each patient. The study was conducted in accordance with the Declaration of Helsinki and the Guidelines for Good Clinical Practice.

### 2.2 Sample collection and plasma preparation

We prospectively collected plasma before ALK TKI therapy, after 2 months of TKI therapy, and at disease progression. Peripheral blood was collected into a 10‐ml K2‐EDTA vacutainer (Becton Dickinson) and centrifuged at 1500× *g* for 15 min within 2 h of collection. The supernatants were sequentially centrifuged at 13,000× *g* for 10 min to remove residual blood compartments. The supernatant was transferred to a 1.5 ml E‐tube and stored at −80°C until use (Figure S1A).

### 2.3 Cell‐free plasma DNA‐NGS test

Plasma samples were retrospectively analyzed using the Guardant360 test (Guardant Health). Single nucleotide variants (SNVs), insertions, and deletions (indel) were assessed in 74 genes (Figure S1B); rearrangements and copy number alterations were evaluated in selected genes according to the manufacturer. All the detected variants were analyzed by Guardant360 bioinformatic pipeline. After excluding the germline variants that were confirmed by the pipeline, somatic variants were considered as circulating tumor DNA (ctDNA).^23^

### 2.4 Statistical analysis

Statistical analyses were performed using Prism software version 9.0 (GraphPad). The nonparametric Mann–Whitney *U*‐test was used to compare two groups. Paired values were compared using the nonparametric Wilcoxon matched‐pairs signed‐rank test. For survival analysis, log‐rank (Mantel‐Cox) test was performed. The hazard ratio for group comparison was calculated by Mantel–Haenszel test. Hazard ratios and corresponding 95% confidence intervals were calculated using the Cox proportional hazards model. We consider *p* values <0.05 as statistically significant. Correlations were analyzed by Spearman's rank correlation coefficient. All tests were the two‐sided test.

## 3 RESULTS

### 3.1 Patient characteristics

Ninety‐two patients were enrolled between April 2015 and July 2019. Baseline patients' clinical characteristics are summarized in Table 1. The median age was 55 (range, 21–79), and 70% were female. About two‐thirds of the patients (68.5%) were never smokers. Eighty‐eight patients (95.7%) had adenocarcinomas, three patients (3.3%) had squamous cell carcinomas, and one patient (1.1%) had neuroendocrine carcinoma. Seventy‐five patients (81.5%) had metastatic disease and 17 patients (18.5%) had recurrent disease. Thirty‐one patients (33.7%) had brain metastases at baseline assessment. Eighty‐one patients (88.0%) received ALK TKI as first‐line (crizotinib, *n* = 59; alectinib, *n* = 22), nine patients (9.8%) received TKI as second‐line (alectinib, *n* = 5; crizotinib, *n* = 2; ceritinib, *n* = 1; brigatinib, *n* = 1), and two patients (2.2%) were treated as third‐line (alectinib, *n* = 1; lorlatinib, *n* = 1) (Table S1).

**TABLE 1:** Patient characteristics

Total	*N* = 92
Age, year	
Range	21–79
Median	55
Gender, *n* (%)	
Female	62 (67.4)
Male	30 (32.6)
Smoking history, *n* (%)	
Current	18 (19.6)
Former	11 (12.0)
Never	63 (68.5)
Tumor histology, *n* (%)	
Adenocarcinoma	88 (95.7)
Squamous cell carcinoma	3 (3.3)
Neuroendocrine carcinoma	1 (1.1)
Stage of the disease, *n* (%)	
Metastatic	75 (81.5)
Recurrent	17 (18.5)
Brain metastases, *n* (%)	
Yes	31 (33.7)
No	61 (66.3)
Prior ALK TKI treatments, *n* (%)	
0	81 (88.0)
≥1	11 (12.0)
ALK TKI, *n* (%)	
Crizotinib	61 (67.3)
Alectinib	28 (30.4)
Brigatinib	1 (1.1)
Ceritinib	1 (1.1)
Lorlatinib	1 (1.1)

Among the enrolled 92 patients, six patients showed early progression and death within 2 months (Figure S2). At the cutoff date of April 20, 2020, the median duration of follow‐up was 19.2 months (range, 0.2 to 60.6). Thirty patients (32.6%) continued to receive ALK TKI without progression and 62 patients (67.4%) demonstrated disease progression.

### 3.2 Genomic profiles by cfDNA‐NGS in ALK positive NSCLC patients at baseline

**FIGURE 1:**
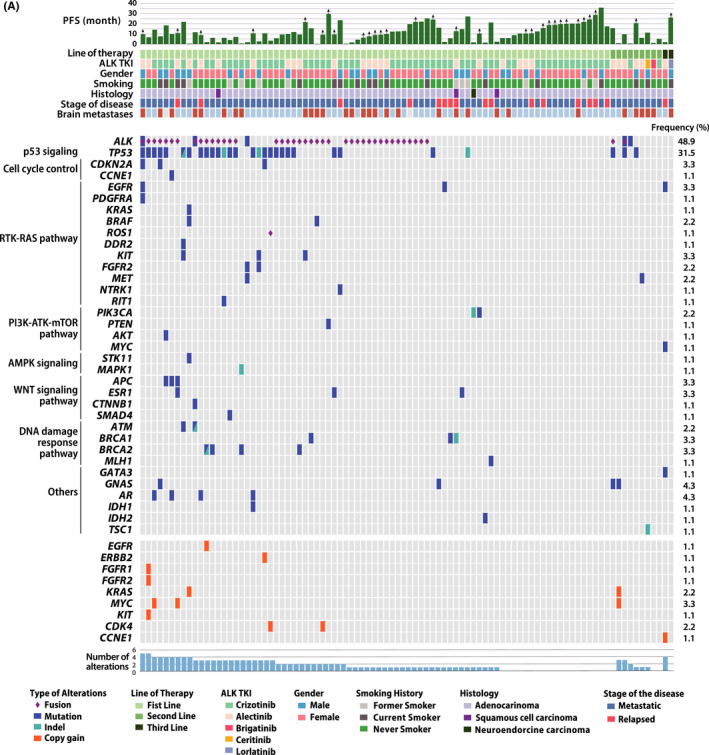
The landscape of somatic alterations in cfDNA‐NGS in *ALK* positive NSCLC. Heatmap of the genomic landscape of ctDNA and clinicopathologic characteristics of patients with *ALK* positive NSCLC matched with Swimmer's plot for progression‐free survival

Comprehensive genomic profiles were analyzed across 92 *ALK* positive NSCLC patients using cfDNA‐NGS at baseline (Figure 1). Among 409 different alterations identified from the cfDNA‐NGS at baseline, 205 (50.1%) were annotated as somatic alteration. There was no correlation between the concentration of cfDNA and the age of patients (Spearman correlation, *r* = −0.03048, *p* = 0.7730) (Figure S3). Cell‐free circulating tumor DNA (ctDNA), which is annotated as somatic alteration in cfDNA‐NGS, was detected in 69 baseline samples (75%). Among 74 genes covered by Guardant360, *ALK* alterations (fusion or mutation) were the most common (48.9%) followed by TP53 alterations (mutation or indel) (31.5%). Other frequent somatic mutations include *GNAS*, *AR*, *CDKN2A*, *EGFR*, *KIT*, *APC*, *ESR1*, *MYC*, *BRCA1*, and *BRCA2*, fusion in *ROS1*, and copy number gains in *ERBB2* and *MYC* which accounts for less 5%.

### 3.3 Detection of ALK alterations by cfDNA‐NGS in ALK positive NSCLC patients at baseline

To evaluate the diagnostic performance of cfDNA‐NGS, we analyzed the detection rate of *ALK* alterations by cfDNA‐NGS in the *ALK* rearranged NSCLC patients who had been diagnosed by ALK IHC, FISH, or targeted NGS from tumor tissue.

**FIGURE 2:**
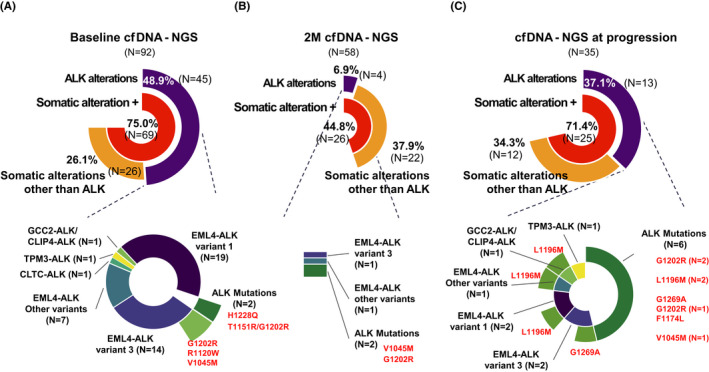
Longitudinal analysis of cfDNA‐NGS in *ALK* positive NSCLC patients who were receiving ALK tyrosine kinase inhibitors. Plasma specimens were collected before ALK TKI treatment start, 2 months after ALK TKI treatment, and at progression. Ninety‐two baseline sample, 58 2‐month follow‐up sample, and 35 at‐progression sample were analyzed by cfDNA‐NGS. The result of cfDNA‐NGS analysis was clustered into three groups; ALK alterations, somatic alterations other than ALK, and no somatic alteration. (A–C) Pie charts demonstrate the frequencies of patients by groups and more detailed information of *ALK* fusions or *ALK* acquired mutations across serial cfDNA‐NGS (A, before ALK TKI treatment, B, 2‐month follow‐up and C, at progression)

Among 69 patients with detectable ctDNA at baseline, 40 patients had *ALK* fusion without *ALK* mutation, three patients had *ALK* fusion with *ALK* mutation, and two patients had *ALK* mutation without *ALK* fusion (Figure 2A). There was no significant correlation or pattern between the detection of ALK alteration in cfDNA‐NGS and the type of ALK test at diagnosis (Table S2). Fusions included *EML4‐ALK* v1 (*n* = 19), *EML4‐ALK* v3 (*n* = 14), *CLTC‐ALK* (*n* = 1), *TPM3‐ALK* (*n* = 1), *GCC2‐ALK/CLIP4‐ALK* (*n* = 1), and other *EML4‐ALK* fusions (*n* = 7). A patient with *EML4‐ALK* v3 and *ALK* V1045M (SMC‐068), a patient with *EML4‐ALK* v3 and *ALK* R1120W (SMC‐092) and a patient with *ALK* H1228Q mutation (SMC‐017) had no history of prior ALK TKI therapy. On the other hand, one patient (SMC‐051) who demonstrated *EML4‐ALK* variant 3 fusion with G1202R mutation had received crizotinib for 39.2 months, and the other patient (SMC‐089) with *ALK* G1202R and T1151R without *ALK* fusion received ceritinib for 35.2 months before study enrollment.

### 3.4 cfDNA‐NGS during ALK TKI therapies in patients with ALK positive NSCLC

Plasms samples were collected at 2 months of ALK TKI treatment and at the time of progression for longitudinal monitoring. Ten out of 96 patients had progressed within 2 months after enrollment. Among 86 patients, 58 patients were analyzed for cfDNA‐NGS at 2 months after ALK TKI treatment (Figure S2). Twenty‐six patients (44.8%) revealed detectable ctDNA, where 22 patients (84.6%) demonstrated somatic alterations other than *ALK*, and four patients (15.4%) demonstrated *ALK* alterations, including *EMK4*‐*ALK* variant 3 (*n* = 1), *EML4*‐*ALK* other variants (*n* = 1), V1045M (*n* = 1), and G1202R (*n* = 1) (Figure 2B).

At the data cutoff, 60 patients (65.2%) had progressed on the ALK TKI therapy. Among 35 (58.3%) samples analyzed for cfDNA‐NGS at progression, 25 samples (71.4%) had detectable ctDNA, where 13 samples (37.1%) had *ALK* alterations, and 12 samples (34.3%) had somatic alterations other than *ALK*. In patients with detectable ctDNA, 52.0% of the patients (*n* = 13) had *ALK* alterations. Three patients had *ALK* fusions without *ALK* mutations (*EML4*‐*ALK* variant 1 (*n* = 1), *EML4*‐*ALK* variant 3 (*n* = 1), and *TPM3*‐*ALK* (*n* = 1)), four patients had *ALK* fusions with *ALK* mutations (*EML4‐ALK* variant 1 with L1196M mutation (*n* = 1), *EML4*‐*ALK* variant 3 with G1269A mutation (*n* = 1), *TPM3*‐*ALK* with L1196M mutation (*n* = 1), and both *GCC2*‐*ALK* and *CLIP4*‐*ALK* with L1196M mutation (*n* = 1)), and six patients had *ALK* mutations without *ALK* fusions (G1202R (*n* = 2), L1196M (*n* = 2), V1045M (*n* = 1), and co‐occurrence of G1269A, G1202R, and F1174L (*n* = 1)) (Figure 2C).

### 3.5 Longitudinal transitions of genomic alterations using cfDNA‐NGS during therapy in ALK positive NSCLC patients

We traced the longitudinal transition of genomic alterations in the cfDNA‐NGS analysis at baseline, 2‐month follow‐up, and progression from the *ALK positive* NSCLC patients during TKI treatment (Figure S4). Patients were clustered into four categories according to cfDNA‐NGS results; *ALK* alterations, somatic alterations other than *ALK*, no somatic alterations, and no cfDNA detected (neither germline nor somatic alteration). Most patients with no somatic alterations at baseline (15 out of 21) continued to show no somatic alterations at 2 months. In contrast, patients with *ALK* alterations at baseline evolved into variable categories at 2 months; no somatic mutations (*n* = 13), somatic alterations other than *ALK* (*n* = 9), *ALK* alterations (*n* = 4), and no ctDNA detected (*n* = 2). Of note, a substantial proportion of (20 of 29) the patients representing no somatic alterations at 2 months continued ALK TKI treatment without progression. Further, two‐third of patients (20 of 30) receiving ALK TKI without progression had revealed no somatic alterations at 2 months.

### 3.6 Associations of ctDNA detection at baseline and 2 months after TKI treatment with clinical outcomes in ALK positive NSCLC patients

**FIGURE 3:**
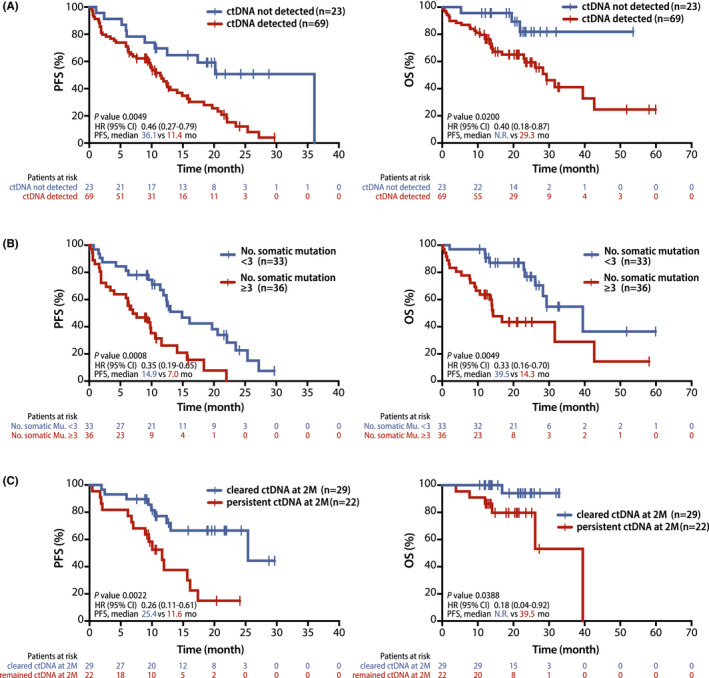
Survival analysis in the patients with *ALK* positive NSCLC according to longitudinal analysis of cfDNA‐NGS. Kaplan–Meier curves for progression‐free survival and overall survival of *ALK* positive NSCLC patients according to the detection of ctDNA at baseline (A), the number of somatic alterations at baseline (B), and detection of ctDNA at 2‐month follow‐up (C)

The absence of detectable ctDNA at baseline (*n* = 23) was associated with significantly longer progression survival (PFS) (median 36.1 vs. 11.4 months, the Hazard ratio (HR) 0.46, 95% confidence interval (CI) 0.27–0.79, *p* = 0.0049) and overall survival (OS) (median not reached vs. 29.3 months, HR 0.40, 95% CI 0.18–0.87, *p* = 0.0200) than those with detectable ctDNA (Figure 3A). Multivariate Cox regression modeling identified the presence of ctDNA at baseline as an independent negative prognostic factor for PFS (HR 2.26, 95% CI 1.12–4.55, *p* = 0.023) and OS (HR 3.33, 95% CI 1.00–11.02, *p* = 0.049) (Figure S5). In subgroup analysis of the patients who received crizotinib as first‐line TKI, the absence of detectable ctDNA at baseline (*n* = 16) was also associated with significantly longer PFS (median 36.1 vs. 10.6 months, HR 0.37, 95% CI 0.20–0.69, *p* = 0.0019) and OS (median not reached vs 28.3 months, HR 0.40, 95% CI 0.15–0.90, *p* = 0.0282) than those with detectable ctDNA (Figure S6A). Moreover, patients with higher number of somatic mutations (≥3, median) (median) at baseline (*n* = 33) showed significantly shorter PFS (7.0 vs. 14.9 months, HR 0.35, 95% CI 0.19–0.65, *p* = 0.0008) and OS (14.3 vs. 39.5 months, HR 0.33, 95% CI 0.16–0.70, *p* = 0.0036) than those with the lower number of somatic mutations (<3, median) (Figure 3B).

Given the possible association of ctDNA concentration with tumor burden,^24^, ^25^ the concentration of ctDNA in plasma was estimated by multiplying the concentration of cfDNA in plasma and the sum of mutation allele fraction that annotated from cfDNA‐NGS analysis algorithm.^26^ Among 69 patients with detectable ctDNA, the median of ctDNA concentration was 69.9 pg/ml (range 3.2–35071.2 pg/ml). Patients with higher ctDNA concentration (>69.9, median) at baseline (*n* = 35) were associated with a trend toward to shorter PFS (9.9 vs. 14.1 months, HR 0.60, 95% CI 0.34–1.08, *p* = 0.0880) but significantly shorter OS (16.8 vs. 39.5 month, HR 0.47, 95% C.I. 0.21–0.98, *p* = 0.0437) than those with lower ctDNA concentration (*n* = 34) (Figure S6B).

Patients with clearance of ctDNA at 2 months (*n* = 29) had significantly longer PFS (median 25.4 vs. 11.6 months, HR 0.26, 95% CI 0.11–0.61, *p* = 0.0022) and significantly longer OS (not reached vs. 26.1 months, HR 0.18, 95% C. 0.04–0.92, *p* = 0.0388) than those without clearance (*n* = 22) regardless of type of ALK TKIs (Figure 3C).

### 3.7 Co‐occurrence of TP 53 alterations was associated with poor outcomes in ALK positive NSCLC

**FIGURE 4:**
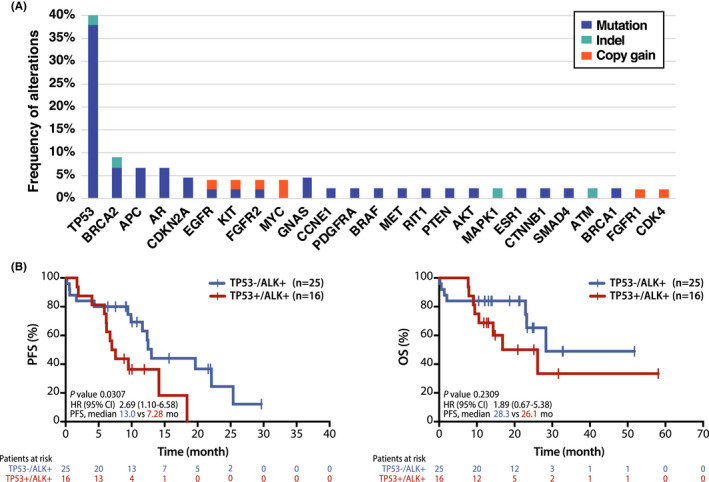
Survival analysis according to co‐occurrence of *TP53* alteration in the patients with *ALK* positive NSCLC. (A) Frequencies of co‐occurring alteration among *ALK* positive NSCLC patients (N = 45) (B) Kaplan–Meier curves for progression‐free survival and overall survival of *ALK* positive NSCLC patients under first‐line ALK TKI according to the co‐occurrence of *TP53* mutation with *ALK* alteration

*TP53* alteration (mutation or indel) (40.0%) was the most frequent co‐occurrent alteration among the patients with ALK‐rearranged NSCLC (Figure 4A). Other frequent somatic mutations, such as *BRCA2*, *APC*, and *AR* were also found.

Patients with co‐occurring *TP53* alterations with *ALK* fusions at baseline (*n* = 16) showed shorter PFS for first‐line ALK TKI (7.28 vs. 13.0 month, HR 2.69, 95% CI 1.10–6.58, *p* = 0.0307) than those without *TP53* alteration (*n* = 25). However, there is no difference in OS according to *TP53* alterations (26.1 vs. 28.3 month, HR 1.89, 95% CI 0.67–5.38, *p* = 0.2309) (Figure 4B).

Considering the biological heterogeneity according to the type of *TP53* mutation,^27^ we mapped the type and location of the *TP53* alterations from the 16 patients with co‐occurring TP53 alterations and ALK fusions in baseline cfDNA (Figure S7A). Fifteen patients with missense mutations (62.5%), five patients with nonsense mutations (31.3%), and one patient with a frameshift mutation (6.25%) were identified. In subgroup analysis, type of *TP53* mutation was not significant factor to demonstrate the differences of PFS in comparing *TP53* wild type to *TP53* mutant (Figure S7B–D).

### 3.8 Resistance mechanisms identified by cfDNA‐NGS at the time of progression in ALK positive NSCLC treated with ALK TKI

Eight patients developed *ALK* resistance mutations after crizotinib therapy: L1196M (*n* = 5), G1269A (*n* = 1), G1202R (*n* = 1), and co‐occurring F1174L, G1202R, and G1269A (*n* = 1). Two patients developed *ALK* resistance mutations after ceritinib: G1202R (*n* = 1), and co‐occurring G1202R and T1151R (*n* = 1). Among 35 patients who performed cfDNA‐NGS at progression, patients with detectable ctDNA (*n* = 25) showed significantly shorter post‐progression survival than those without detectable ctDNA (*n* = 10; 16.5 vs. undefined months, HR 3.43, CI 1.13–10.37, *p* = 0.0291) (Figure S8C).

### 3.9 Activation of the bypass signaling was identified by longitudinal monitoring cfDNA‐NGS in ALK positive NSCLC patients who were resistant to ALK TKI

**FIGURE 5:**
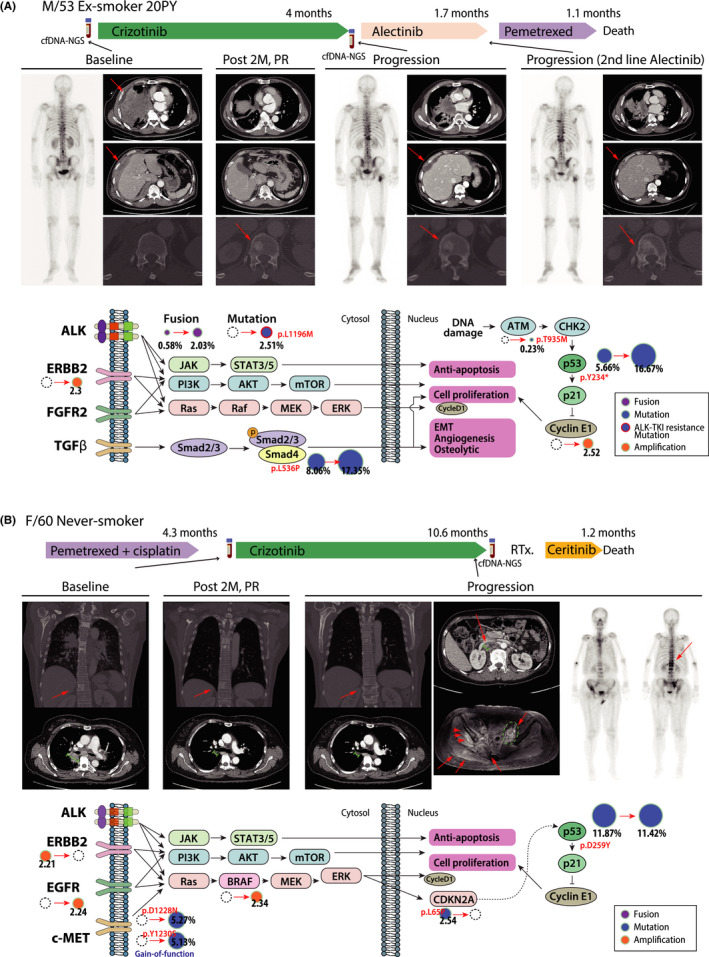
Patients who were annotated bypass signaling activation by cfDNA‐NGS monitoring showed resistance to ALK‐TKI. (A, B) The treatment summary is displayed on the upper panel. The images of whole‐body bone scan and enhance computerized tomography (CT) during treatment are displayed on the middle panel. A diagram of the signaling pathway demonstrates the principal changes between pretreatment and at progression in cfDNA‐NGS and the resistance mechanisms of ALK acquired resistance mutation and bypass signaling activation. The size of the circle represents allele frequency (%) of fusion, indel or mutation, or copy number

Matching analysis of genomic alterations by cfDNA‐NGS between baseline and at progression identified not only ALK mutation but also bypass signaling activation. A 53‐year‐old patient (SMC‐036) initially presented lung, pleural, and bone metastases showing robust tumor shrinkage with crizotinib. However, after 4 months of crizotinib, progression occurred. Compared to baseline cfDNA‐NGS results L1196M mutation along other genomic alterations *ERBB2* copy number amplification and *CCNE1*gene amplification were newly identified at progression. Further, the ctDNA percentage of *SMAD4* p.L536P mutation (from 8.06% to 17.35%) and *TP53* p.Y234* was increased at progression. After progression, patient received alectinib but response was observed only in lung and pleura but progressed in the bone. Finally, patient died shortly of disease progression after chemotherapy. (Figure 5A).

Another patient who did not reveal *ALK* alterations in ctDNA showed bypass pathway activation in the analysis of cfDNA‐NGS at progression, which was suggestive of resistance mechanism against ALK TKI (Figure 5B). A 60‐year‐old woman (SMC‐20) showed a partial response to crizotinib. At progression, no ALK mutation was noted but activation of c‐met pathway including *MET* Y1230 (or Y1248) (p.Y1230S, NM_000245.2:c.3689A > C, 5.13%) mutation and *MET* D1228N (or D1246N) (p.D1228N, NM_000245.2:c.3682G > A, 5.27%), which are located on kinase domain of MET along with amplification of *BRAF*. This patient experienced rapid progression to ceritinib as salvage therapy.

## 4 DISCUSSION

In this study, we prospectively collected serial plasma samples from 92 patients with ALK rearranged NSCLC who were treated with ALK TKIs and performed comprehensive genomic analysis using cfDNA‐NGS.

We found that ctDNA was detected in 75% of baseline samples and among these 62.3% of *ALK* fusions and 2.8% of *ALK* mutations without fusion were identified by cfDNA‐NGS, which is consistent with previous studies.^17^, ^19^, ^21^ Further, cfDNA‐NGS was able to detect not only *EML4‐ALK* variants but also various fusion partners of *ALK* fusion, suggesting high performance of NGS with liquid biopsy.^18^, ^20^, ^21^

Our data represent the landscape of genomic alterations in ctDNA from TKI‐naïve *ALK* positive NSCLC patients. At baseline, cfDNA‐NGS can detect not only *ALK* gene alterations, but also other coexisting somatic mutations *TP53*, *CDKN2A*, *EGFR*, etc. and copy number variants like *ERBB2* and *MYC*, although the clinical significance of these co‐occurring alterations should be further determined. Most of previous studies with cfDNA were focused to detect *ALK* resistance mutations in *ALK* positive NSCLC patients treated with ALK TKI as second‐line therapy.^17^, ^18^, ^19^ The proportion of treatment naïve *ALK* positive NSCLC patients was less than 50% and small number of patients were evaluated. In contrast, 75 of 92 (81%) patients enrolled in this study were ALK TKI naïve status. Moreover, the plasma samples were prospectively collected, and a large number of patients were longitudinally monitored. Further, with long term follow‐up, we can evaluate the role of cfDNA‐NGS analysis as predictive and prognostic value in *ALK* positive NSCLC patients treated with TKI.

We also found that the absence of detectable ctDNA at baseline was associated with longer PFS and OS. Further, patients with clearance of ctDNA at 2 months after ALK TKIs showed significantly longer PFS and OS that those without clearance. These results confirmed previous studies,^28^, ^29^ suggesting that patients with non‐shedder *ALK* positive NSCLC exhibit less aggressive tumor biology. Besides, patients without detectable ctDNA at baseline were likely to show undetectable ctDNA at 2 months after TKI therapy (16/23) and to demonstrate durable ALK TKI response (12 ongoing therapies among 23 patients). However, due to small number of progression event, the response of ALK‐TKI did not show any significant correlation either the presence of ctDNA at baseline (Table S3) or at 2 months (Table S4). As far as we know, this is the first report that demonstrates the predictive value of cfDNA‐NGS in *ALK* positive NSCLC based on the PFS and OS data.

At progression, cfDNA‐NGS detected various *ALK* mutations including L1196M, G1269A, G1202R, F1174L, and T1151R which are known to be resistance to ALK TKIs. Since the spectrum of resistance *ALK* mutation becomes more complex with sequential use of ALK TKIs or upfront use of second or third generation ALK TKIs, the identification of resistance *ALK* mutation would be more critical. Tissue biopsy is considered golden standard to elucidate the genomic alterations but the acquisition of adequate tissue at the time of progression is more challenging. Although patients were not treated with other ALK inhibitors according to the *ALK* mutation status at progression because specimens were retrospectively analyzed in our study, these different *ALK* mutations might guide treatment decision given the differential sensitivity to the type of ALK TKIs. Given that, cfDNA‐NGS is reasonable approach in clinical practice as alternative measure to detect *ALK* resistance mutation especially in case of sequential treatment of ALK TKIs.

It has been reported that coexistence of genomic alterations such as *TP53* is associated with poor outcome in oncogenic driver in NSCLC.^30^ In this study, patients with co‐occurring *TP53* alterations and *ALK* fusions at baseline were associated with shorter PFS and OS than those without *TP53* alterations, which is consistent with previous results.^31^ In subgroup analysis for *TP53* missense mutation and nonsense mutation, while subtype showed trends for shorter PFS compared to *TP53* wild type, there were not significant differences in PFS between *TP53* mutant subtype and *TP53* wild type. Larger number of patients may be necessary for investigating clinical or biological significance according to the type of *TP53* mutation in *ALK* positive NSCLC.

Various agents targeting p53 pathway for cancer therapy are being investigated.^32^ Eprenetapopt, mutant p53 reactivator, demonstrated promising efficacy in *TP53*‐mutant myelodysplastic syndrome or acute myeloid leukemia.^33^ In solid tumor, a phase 1b/2 clinical trial of Eprenetapopt in combination with pembrolizumab is ongoing in patients with advanced or metastatic solid cancer (NCT04383938).^34^ *TP53* mutation was reported as favorable prognostic marker in the patients who treated with immune checkpoint inhibitors (ICIs), and as the association of high tumor mutation burden and activated immune cell infiltration.^35^, ^36^ As *TP53* mutation was associated with higher genetic instability in ALK positive NSCLC,^37^ we identified that the number of detected somatic mutations in cfDNA in the patients with *TP53* mutations was significantly higher than those with *TP53* wild type (Figure S7E). Considering this rationale, ICIs or the combination therapy of *TP53* targeting agent with ICIs can be effective in the *ALK* positive NSCLC patients with co‐occurring TP53 mutations.

To expand PFS and OS in *ALK* positive NSCLC, it is important to identify and target the patient‐specific resistance mechanism of ALK TKIs accurately. Although second biopsy is vital for profiling the resistance mechanism at progression, on‐treatment biopsy is not always applicable due to the limitation of approach or patient's medical condition. Performing cfDNA‐NGS at progression in ALK positive NSCLC could be prognostic and informative. The patients with no somatic alteration detected cfDNA had better post‐progression survival than those with somatic alterations (Figure S6C), so that the physician could have detected and responded to another progression more rapidly. NGS of cfDNA at progression in *ALK* positive NSCLC can guide the choice of ALK TKI based on the profile of *ALK* resistance mutation. From the matching the result of cfDNA‐NGS at progression with subsequent therapies retrospectively, ceritinib was more active for L1196M mutation than alectinib (Figure S8). Further, with comprehensive analysis of genomic alterations using cfDNA‐NGS, activation of other bypass signaling can be identified and compared between baseline and at the time of progression (Figure S8). Representative two cases indicate that the resistance mechanism to ALK TKIs is quite complex and will be variable among patients (Figure 5). For example, in case of SMC‐036, newly identified *ERBB2* amplification along with L1196M after crizoinib might explain why patient was initially well responded to alectinib but experienced progressed shortly. Another case (SMC‐020) who developed c‐met mutation without alteration of ALK mutation did not response to ceritinib, can be attributed to the activation of c‐met pathway. In this case, c‐met inhibitor with second generation of ALK TKI might be beneficial. Taken together, real‐time monitoring of genomic alterations during ALK TKI treatment using cfDNA‐NGS as noninvasive method would be valuable to make treatment decision.

There are limitations to this study. The patients in this study were treated with heterogeneous. As the patients had been enrolled for a long period of time (from April 2015 to July 2019), the patients were treated with crizotinib, alectinib, ceritinib, brigatinib, or lorlatinib that were accessible depending on the period. Patient samples were prospectively collected but analysis was conducted in retrospective manner. Hence, patients were not treated with matched targeted therapy according to the resistance mechanism in real‐time. Since most of patients were treated with crizotinib as first‐line, data are limited for resistance to upfront use of second generation ALK TKIs, where most of patients are ongoing without progression. Further analysis will be available in the future. Nevertheless, to the best of our knowledge, our study is one of the largest prospective study to evaluate the role of cfDNA‐NGS in clinical practice.

In conclusion, NGS of cfDNA is useful not only for the detection of *ALK* fusions and resistance mutations, but also for assessing prognosis and monitoring the dynamic changes of genomic alterations in *ALK* positive NSCLC treated with ALK‐TKI.

## CONFLICT OF INTEREST

S. Olsen is an employee of and holds ownership interest in Guardant Health. M. Lefterova is an employee of Guardant Health. J. Odegaard is an employee of and holds ownership interest in Guardant Health. No potential conflicts of interest were disclosed by the other authors.

## AUTHOR CONTRIBUTIONS

Minsuk Kwon, Steve Olsen, and Myung‐Ju Ahn wrote the manuscript. Steve Olsen and Myung‐Ju Ahn initiated the study concept. Minsuk Kwon, Bo mi Ku, Sehhoon Park, Hyun‐Ae Jung, Jong‐Mu Sun, Se‐Hoon Lee, Jong‐Mu SunA., and Myung‐Ju Ahn analyzed the clinical data. Minsuk Kwon, Bo mi Ku, Martina Lefterova, and Justin Odegaard analyzed the genomic data. Hyun‐Ae Jung, Jong‐Mu Sun, Se‐Hoon Lee, Jong‐Mu SunA., Keunchil Park, and Myung‐Ju Ahn supervised the patient enrollment and participated and handled the study participants. All authors approved the final manuscript.

## Supporting information


Supplementary Figure 1A
Supplementary Figure 1B.
Supplementary Figure 2.
Supplementary Figure 3.
Supplementary Figure 4.
Supplementary Figure 5.
Supplementary Figure 6.
Supplementary Figure 7.
Supplementary Figure 8.

Supplementary table 2

Supplementary table 1
Supplementary Table 3
Supplementary Table 4

## Data Availability

Data sharing is not applicable to this article as no new data were created or analyzed in this study.
