# Supplementary material for: Longitudinal monitoring by next‐generation sequencing of plasma cell‐free DNA in ALK rearranged NSCLC patients treated with ALK tyrosine kinase inhibitors
Source: Cancer Med. 2022 Apr 19;11(15):2944–56. doi: 10.1002/cam4.4663 (PMC9359877; doi:10.1002/cam4.4663)

Supplementary figure 1

A

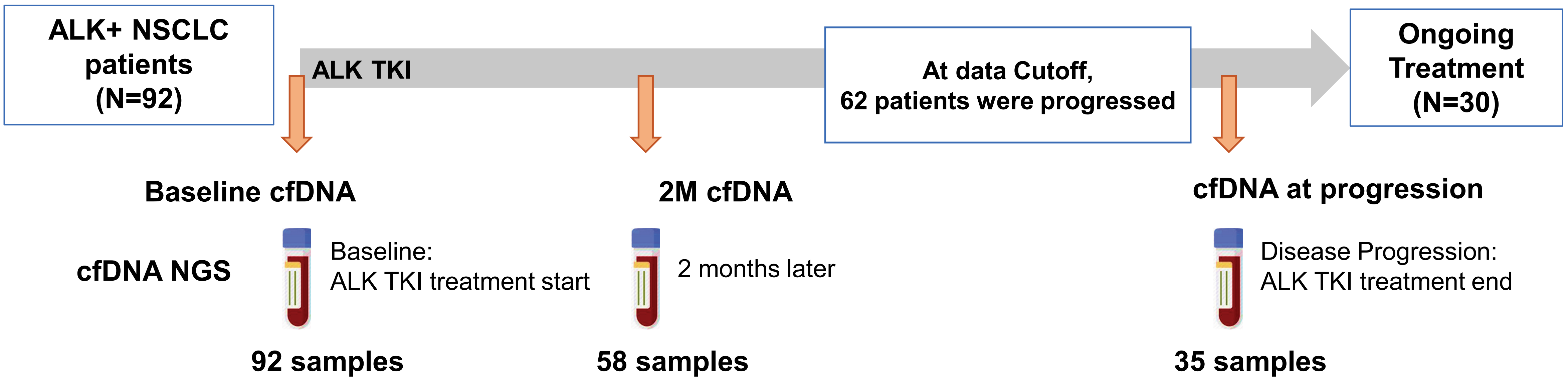

B

Point Mutations (SNVs) (73 Genes)						*Indels (23 Genes)*		*Amplifications (18 Genes)*		*Fusions (6 Genes)*
AKT1	*ALK*	*APC*	*AR*	*ARAF*	*ARID1A*	*ATM*	*APC*	*AR*	*BRAF*	*ALK*
ATM	*BRAF*	*BRCA1*	*BRCA2*	*CCND1*	*CCND2*	*ARID1A*	*BRCA1*	*CCND1*	*CCND2*	*FGFR2*
CCNE1	*CDH1*	*CDK4*	*CDK6*	*CDKN2A*	*CTNNB1*	*BRCA2*	*CDH1*	*CCNE1*	*CDK4*	*FGFR3*
DDR2	*EGFR*	*ERBB2 (HER2)*	*ESR1*	*EZH2*	*FBXW7*	*CDKN2A*	*EGFR*	*CDK6*	*EGFR*	*NTRK1*
FGFR1	*FGFR2*	*FGFR3*	*GATA3*	*GNA11*	*GNAQ*	*ERBB2*	*GATA3*	*ERBB2*	*FGFR1*	*RET*
GNAS	*HNF1A*	*HRAS*	*IDH1*	*IDH2*	*JAK2*	*KIT*	*MET*	*FGFR2*	*KIT*	*ROS1*
JAK3	*KIT*	*KRAS*	*MAP2K1/MEK1*	*MAP2K2/MEK2*	*MAPK1/ERK2*	*MLH1*	*MTOR*	*KRAS*	*MET*	
MAPK3/ERK1	*MET*	*MLH1*	*MPL*	*MTOR*	*MYC*	*NF1*	*PDGFRA*	*MYC*	*PDGFRA*	
NF1	*NPM1*	*NOTCH1*	*NFE2L2*	*NRAS*	*NTRK1*	*PTEN*	*RB1*	*PIK3CA*	*RAF1*	
NTRK3	*PDGFRA*	*PIK3CA*	*PTEN*	*PTPN11*	*RAF1*	*SMAD4*	*STK11*			
RB1	*RET*	*RHEB*	*RHOA*	*RIT1*	*ROS1*	*TP53*	*TSC1*			
SMAD4	*SMO*	*STK11*	*TERT*†	*TP53*	*SC1*	*VHL*				
VHL										

**Exons selected to maximize detection of known somatic mutations / † IncludesTERTpromoter region*

Supplementary figure 2

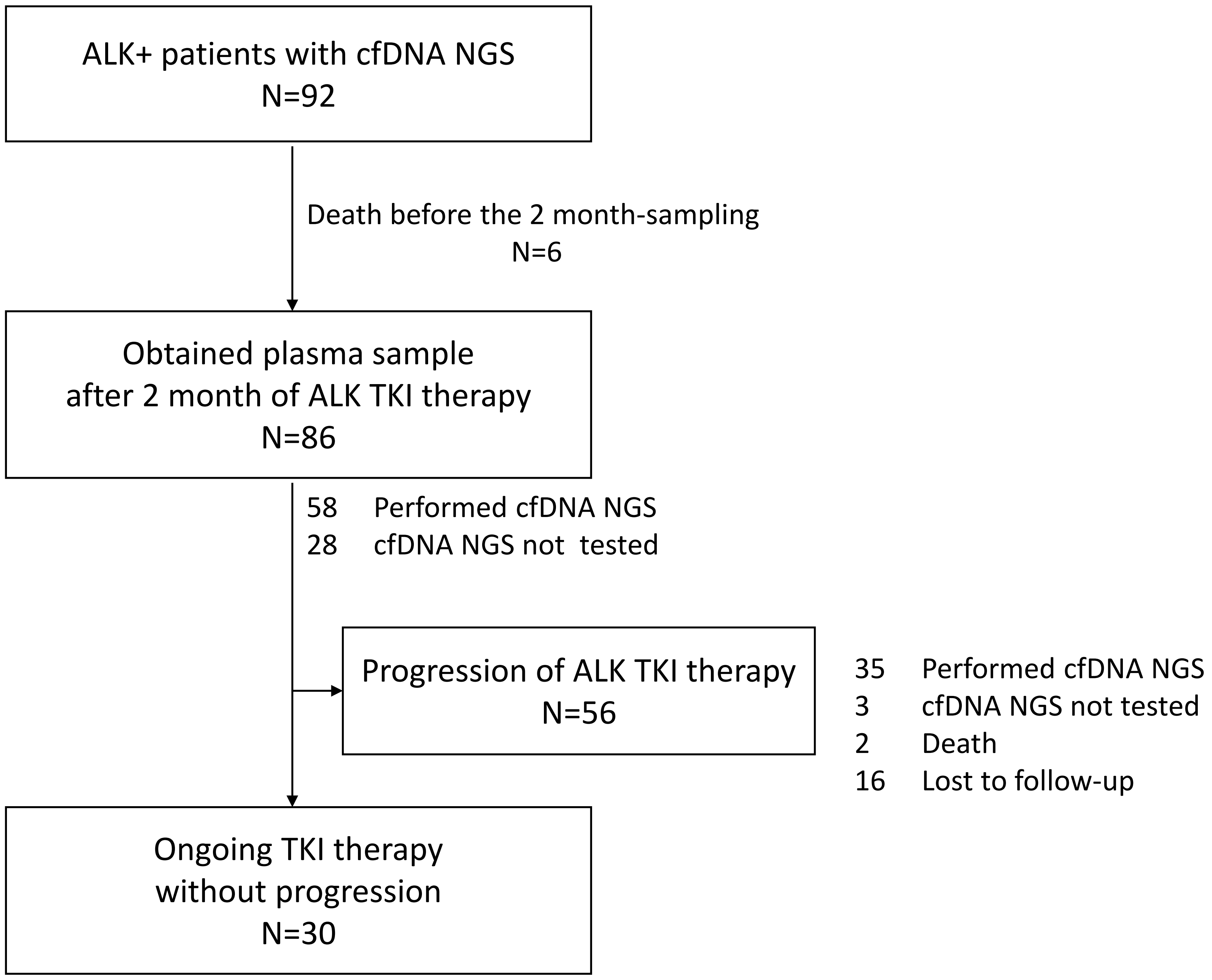

Supplementary figure 3

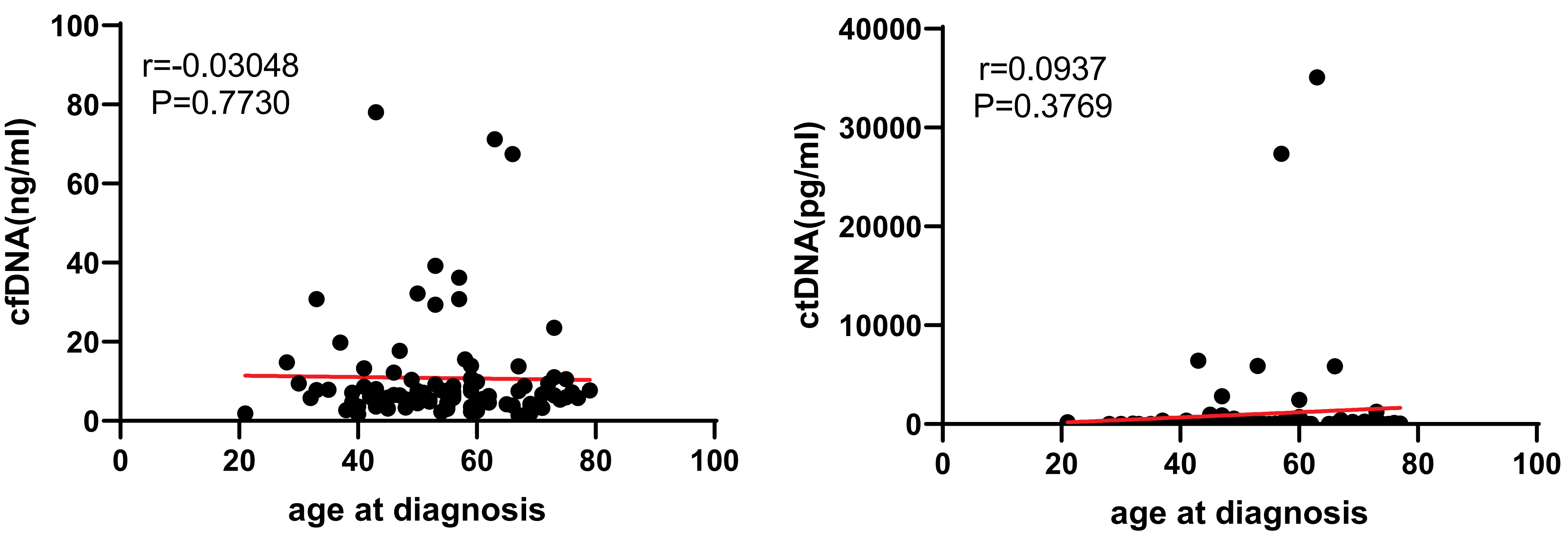

Supplementary figure 4

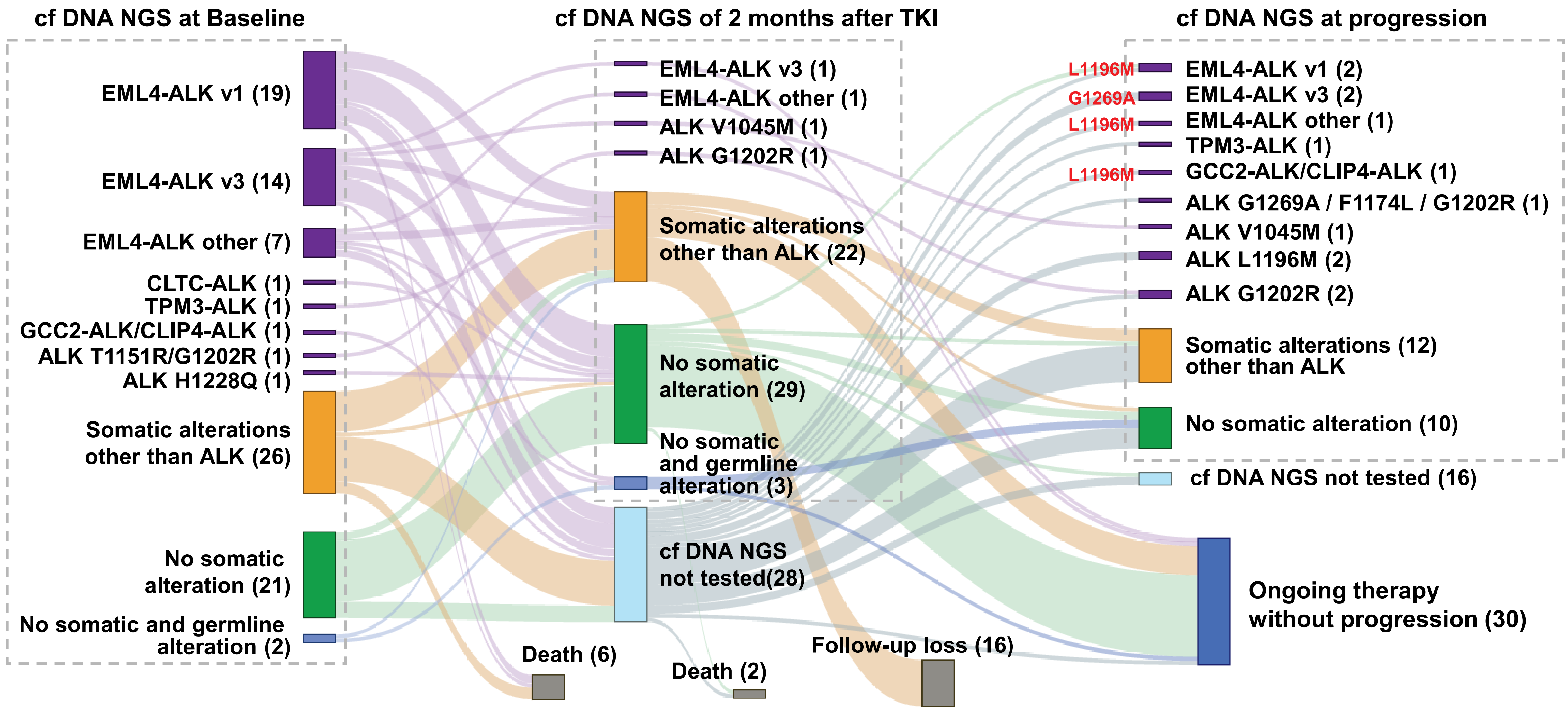

Supplementary figure 5

Variable	HR (95% CI)	P-value	HR (95% CI)	P-value
Age	1.023 (1.003-1.047)	0.047	1.033 (1.003-1.064)	0.031
Brain metastases	0.965 (0.543-1.715)	0.903	0.552 (0.231-1.317)	0.181
Prior systemic therapy	1.144 (0.655-1.998)	0.636	0.659 (0.655-3.877)	0.242
ctDNA detected	2.256 (1.119-4.547)	0.023	3.326 (1.004-11.022)	0.049
≥2nd-line ALK-TKI	1.547 (0.688-3.478)	0.291	2.247 (0.766-6.593)	0.140

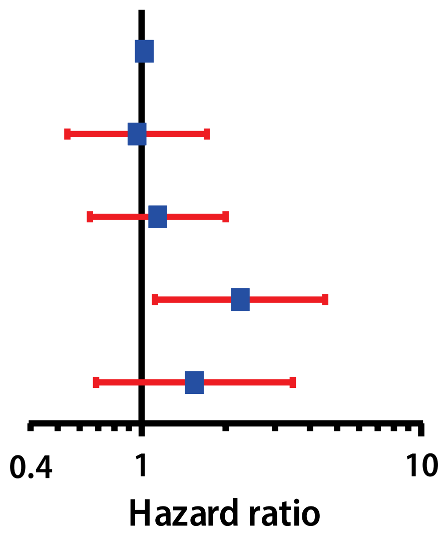

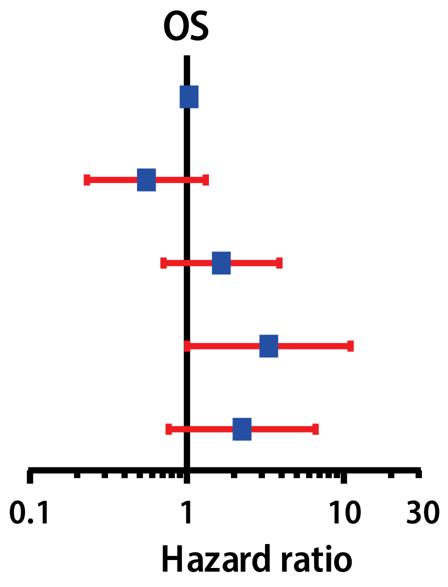

Supplementary figure 6

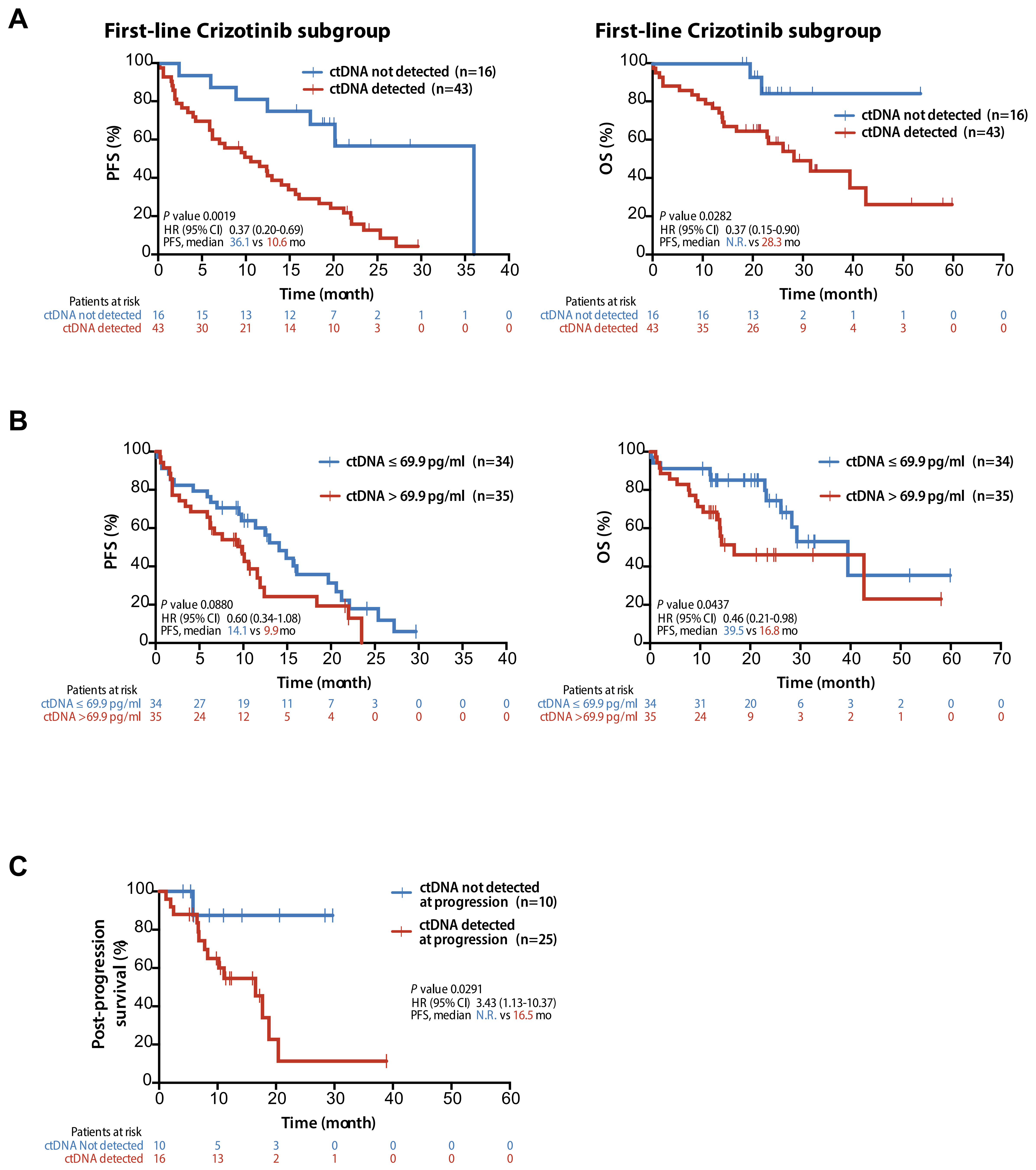

Supplementary figure 7

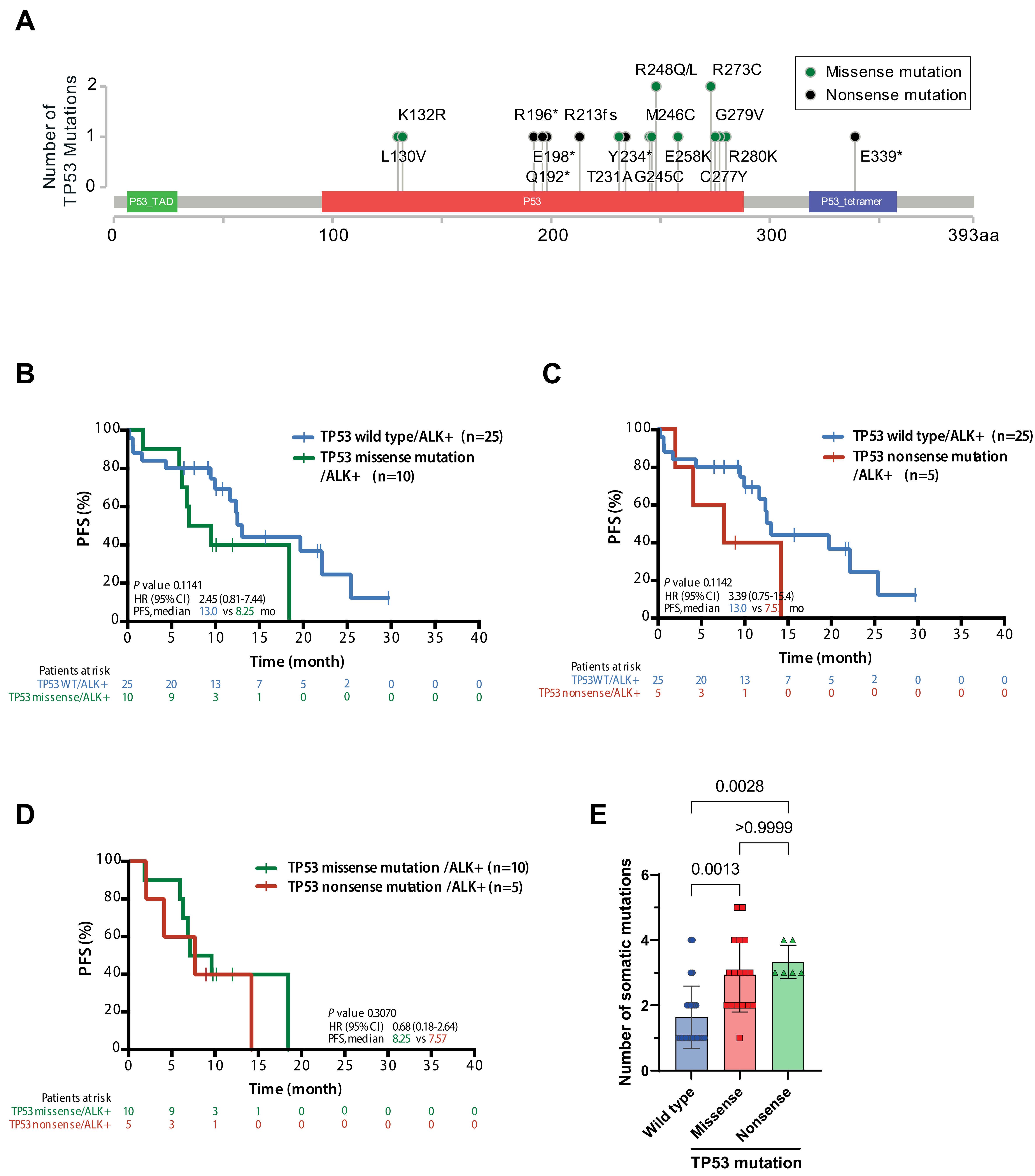

Supplementary figure 8

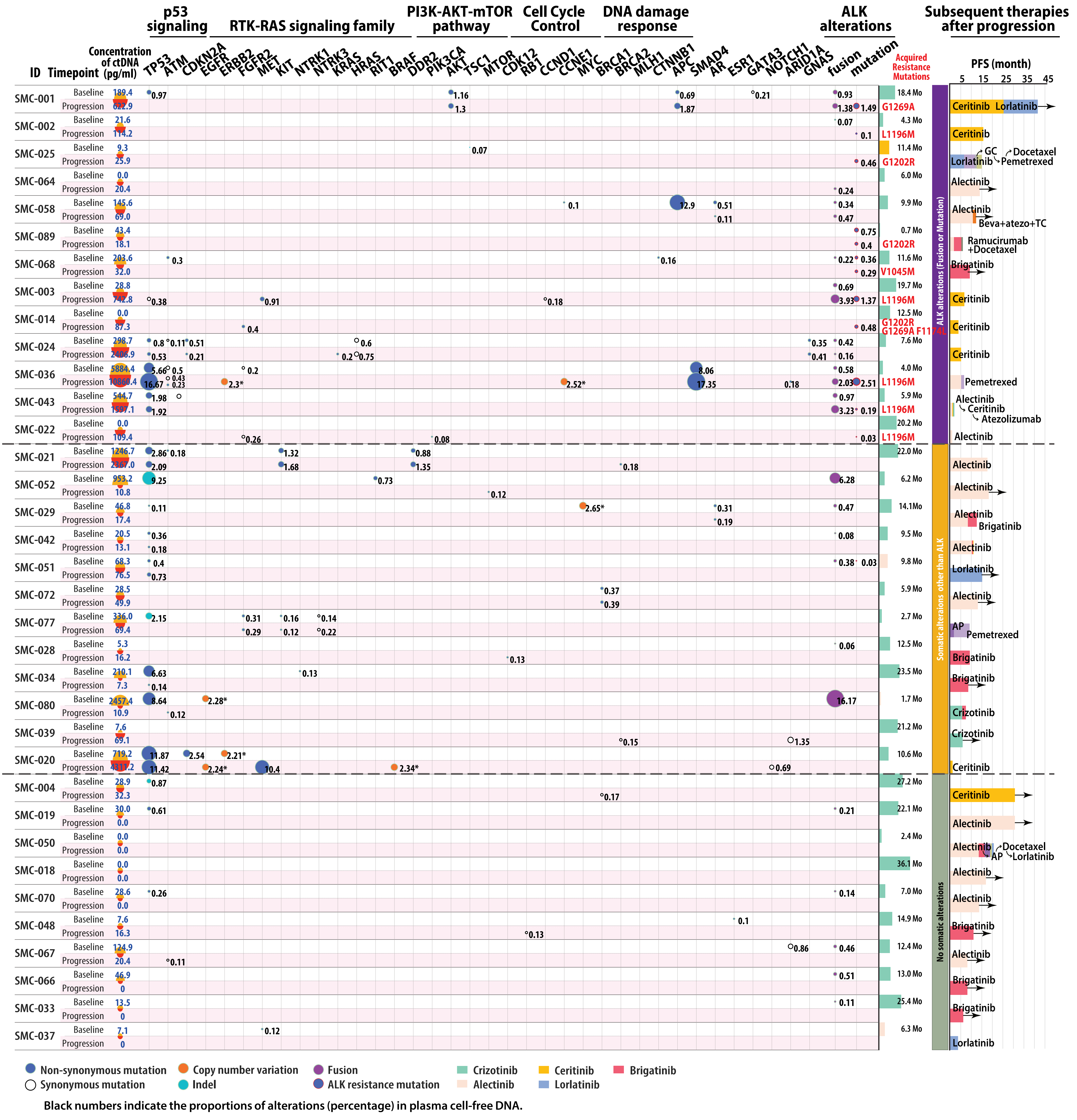

Supplement: Supplementary file 1 — Supplementary Figure 1A Supplementary Figure 1B. Supplementary Figure 2. Supplementary Figure 3. Supplementary Figure 4. Supplementary Figure 5. Supplementary Figure 6. Supplementary Figure 7. Supplementary Figure 8. [file CAM4-11-2944-s001.pdf]
