# Supplementary material for: Longitudinal monitoring by next‐generation sequencing of plasma cell‐free DNA in ALK rearranged NSCLC patients treated with ALK tyrosine kinase inhibitors
Source: Cancer Med. 2022 Apr 19;11(15):2944–56. doi: 10.1002/cam4.4663 (PMC9359877; doi:10.1002/cam4.4663)
Supplement: Supplementary file 3 — Supplementary table 1 Supplementary Table 3 Supplementary Table 4 [file CAM4-11-2944-s003.docx]

**Supplementary table 1. ALK TKIs treated as the first, or second or more line**

|  | **First line**  **(*N*=81)** | **Second line**  **(*N*=9)** | **Third line**  **(*N*=2)** | **Total**  **(*N*=92)** |
| --- | --- | --- | --- | --- |
| **Crizotinib** | **59** | **2** | **0** | **61** |
| **Alectinib** | **22** | **5** | **1** | **28** |
| **Brigatinib** | **0** | **1** | **0** | **1** |
| **Ceritinib** | **0** | **1** | **0** | **1** |
| **Lorlatinib** | **0** | **0** | **1** | **1** |

**Supplementary table 3. Response of ALK TKIs according to the presense of ctDNA at baseline in cfDNA-NGS**

| **Best response** | **ctDNA not detected**  **(*N*=23)** | **ctDNA detected**  **(*N*=69)** | ***P*-value** |
| --- | --- | --- | --- |
| **Partial response** | **19 (86.4%)** | **54 (78.3%)** |  |
| **Stable disease** | **2 (9.1%)** | **6 (8.7%)** |  |
| **Disease progression** | **1 (4.5%)** | **9 (13.0%)** |  |
| **Not evaluable** | **1** | **0** |  |
| **Objective response rate** | **86.4%** | **78.3%** | **0.5451*** |
| **Disease control rate** | **95.5%** | **87.0%** | **0.4409*** |

* Fischer’s exact test

**Supplementary table 4. Response of ALK TKIs according to the presense of ctDNA at 2 months in cfDNA-NGS**

| **Best response** | **cleared ctDNA**  **at 2 months** | **Persistent ctDNA**  **at 2 months** | ***P*-value** |
| --- | --- | --- | --- |
| **Partial response** | **26 (81.3%)** | **20 (76.9%)** |  |
| **Stable disease** | **2 (6.3%)** | **0 (0%)** |  |
| **Disease progression** | **4 (12.5%)** | **6 (23.1%)** | **0.463*** |
| **Objective response rate** | **81.3%** | **76.9%** | **0.752*** |

* Fischer’s exact test
